# Cigarette Smoking Is Significantly Linked to Sexual Dissatisfaction in Chinese Heroin-Dependent Patients Receiving Methadone Maintenance Treatment

**DOI:** 10.3389/fpsyt.2019.00306

**Published:** 2019-05-22

**Authors:** Bao-Liang Zhong, Yan-Min Xu, Wu-Xiang Xie, Jin Lu

**Affiliations:** ^1^Research Center for Psychological and Health Sciences, China University of Geosciences, Wuhan, China; ^2^Affiliated Wuhan Mental Health Center, Tongji Medical College of Huazhong University of Science and Technology, Wuhan, China; ^3^Peking University Clinical Research Institute, Peking University Health Science Center, Beijing, China; ^4^Department of Psychiatry, The First Affiliated Hospital of Kunming Medical University, Kunming, China

**Keywords:** smoking, sexual dysfunction, heroin dependence, methadone maintenance treatment

## Abstract

**Background:** Cigarette smoking is associated with sexual dysfunction in the general population. Both smoking and sexual dysfunction are common in heroin-dependent patients (HDPs) receiving methadone maintenance treatment (MMT), but their association in MMT HDPs is rarely studied. This study examined the association between smoking and sexual dissatisfaction in Chinese HDPs receiving MMT.

**Methods:** In total, 480 Chinese HDPs, who had sex with their regular or irregular sex partners within one month prior to the study, were included from three MMT clinics in Wuhan, China. Sexual dissatisfaction was assessed with one single question. Socio-demographic and clinical data and smoking characteristics were collected with a standardized questionnaire. Multiple binary logistic regression was used to analyze the association between smoking and sexual dissatisfaction, as well as the associations between levels of smoking and nicotine dependence and sexual dissatisfaction.

**Results:** The prevalence of current smoking was 95.6% in HDPs receiving MMT. Rates of sexual dissatisfaction were higher in current smokers than non-smokers (32.9% vs. 14.3%) with a borderline significant P value of 0.074. After adjusting potential socio-demographic and clinical confounders, current smoking was significantly linked to sexual dissatisfaction (OR = 1.95, P = 0.026), and heavy smoking and severe nicotine dependence were significantly linked to sexual dissatisfaction (OR = 1.80, P = 0.025; OR = 3.27, P < 0.001).

**Conclusion:** Smoking is significantly associated with sexual dysfunction in HDPs receiving MMT. It deserves further investigation as to whether quitting smoking can improve the sexual function of methadone-maintained patients.

## Introduction

Cigarette smoking is a major risk factor for elevated mortality and a variety of physical morbidities, including cardiovascular and respiratory diseases, cancer, and diabetes (1–4). Smoking also adversely affects sexual health. For example, cross-sectional and prospective studies have confirmed the independent contribution of smoking to the presence of sexual dysfunction in both men and women, and there is a dose-response relationship between cigarette exposure and the risk of sexual dysfunction (5–9). Further, growing evidence has shown significant improvement in sexual function as a result of smoking cessation (10–12).

Sexual dysfunction is highly prevalent among heroin-dependent patients (HDPs) receiving methadone maintenance treatment (MMT). For example, 66.3% and 77.5% men under MMT had sexual disorders and erectile dysfunction, respectively, while in women under MMT, 23.1% reported some forms of sexual dysfunction and 22.9% were dissatisfied with their sexual lives (13–15). Empirical studies have demonstrated that sexual dysfunction is one of the most important causes for patients’ early dropout from MMT, poor intimate relationships, and low quality of life (16–18). Due to these reasons, sexual dysfunction has been recognized as a major challenge in the clinical management of methadone-maintained HDPs.

The etiology of sexual dysfunction is complex, in particular regarding the sexual problems of patients diagnosed with heroin dependence and receiving opioid replacement therapy (ORT) (19). Commonly reported risk factors for sexual dysfunction in the general population include old age, unemployment, diabetes, heart disease, urinary tract disorders, depression, obesity, and smoking (1, 20, 21). A few recent studies have examined factors associated with sexual dysfunction in patients receiving ORT and they found that, in addition to some common factors such as old age and poor physical health, treatment-related factors such as opioid maintenance dosage and clinical factors such as pain and sleep disturbance also significantly contributed to sexual dysfunction within this patient population (17, 22–25). Nevertheless, because of the limited number of related studies, risk factors for sexual problems of methadone-maintained patients has not been fully elucidated (22).

Smoking is an extremely common health risk behavior in HDPs receiving MMT, with a prevalence of 87.2%–98.1% (26–28). Given the high prevalence of smoking and sexual dysfunction in methadone-maintained HDPs and the significant link between smoking and sexual dysfunction in the general population, we speculate that smoking is significantly associated with sexual dysfunction of methadone-maintained HDPs. However, to our knowledge, this relationship is rarely studied in MMT patients. Addressing smoking as a preventable and modifiable risk factor is a potentially useful strategy to reduce or relieve the sexual problems of MMT patients. The present study examined the relationship between smoking and sexual dissatisfaction in a sample of Chinese methadone-maintained HDPs.

## Materials and Methods

### Subjects

This study was a secondary data analysis using data from a large-scale cross-sectional survey, which determined the quality of life, mental health, sexual life satisfaction, and non-fatal suicidal behaviors of patients of three MMT clinics in Wuhan, China, from June 2009 to July 2010 (22, 25, 29–31). To be eligible for the current analysis, patients must be 20 years or older, be taking methadone orally at the three MMT clinics, meet DSM-IV diagnostic criteria for lifetime heroin dependence, and must have had sex with their regular or irregular sex partners within one month prior to the survey. We excluded patients with severe physical illnesses, alcohol dependence, organic mental disorders, or psychotic symptoms. During the survey period, a total of 749 patients were receiving treatment at the three MMT clinics and screened for eligibility, of which 519 fulfilled the inclusion criteria and 480 finally provided complete questionnaire data.

The study protocol was approved by the Ethical Review Board of Wuhan Mental Health Center. Declarations of anonymity and confidentiality had been made and all subjects provided written informed consent before the formal survey.

### Instruments and Procedures

We used a standardized self-report questionnaire to collect data. Before the formal study, a pilot study was carried out among a sample of 48 MMT HDPs to test our data collection procedure for feasibility. After the pilot study, necessary amendments were also made to the questionnaire to ensure its acceptability and feasibility.

The formal questionnaire consisted of the following parts: 1) socio-demographics such as sex and age; 2) clinical characteristics including the main route of past heroin administration, length of heroin use, MMT duration, methadone dosage, and depressive symptoms; 3) sexual life satisfaction; and 4) characteristics of smoking including the number of cigarettes smoked daily (NCSD) and nicotine dependence.

Depressive symptoms were assessed with the validated Chinese version of the Zung’s Self-rating Depression Scale (SDS), which has 20 items, each scored on a four-point Likert scale. Its total score ranges from 20 to 80, with a score of 40 and above denoting clinically significant depressive symptoms (32).

Sexual life satisfaction was assessed with a single-item question: “Over the past month, how satisfied have you been with your overall sexual life?” Respondents were asked to rate on a five-point scale (1 = very dissatisfied, 2 = dissatisfied, 3 = fair, 4 = satisfied, and 5 = very satisfied). This simple measure of sexual function has been widely used in existing epidemiological studies of sexual health with satisfactory reliability and validity (15, 33, 34). Unlike other scales, which often assess the sexual function of one sex only, this single item has the advantage of measuring sexual function of both sexes (25). In our pilot study, the validity of this measure was tested by correlating its score with the four subscale scores of the Scale for Quality of Sexual Function. Results showed moderate-to-high correlations between the two scales, indicating that the satisfactory validity of this single-item measure (22, 25). Consistent with prior studies, respondents were categorized as having sexual dissatisfaction if they rated their sexual life as “very dissatisfied” and “dissatisfied.”

In this study, current smokers were defined as those who were currently smoking one or more cigarette a day and had smoked for at least half a year (35). Based on NCSD, current smokers were divided into light (<10/day), moderate (10–19/day), and heavy smokers (>19/day) (35). Nicotine dependence was assessed with the Chinese Fagerstrom Test for Nicotine Dependence (FTND), which consists of six questions (36). The FTND score ranges from 0 to 10, with 0–4, 5, and 6–10 representing mild, moderate, and severe levels of nicotine dependence, respectively (37).

Six treating psychiatrists of the HDPs of the three MMT clinics, were trained to be the survey investigators. These investigators invited eligible patients to join the study, read out questions for patients who had difficulties in completing the questionnaire, and checked answers in the questionnaire individually for logic errors or missing values before collection.

### Statistical Analysis

Prevalence rates of sexual dissatisfaction according to smoking characteristics were calculated. Socio-demographic and clinical characteristics of smokers and non-smokers compared by χ2 test. Because smokers and non-smokers were not comparable in terms of some socio-demographic and clinical variables, the smoking-sexual dissatisfaction association was tested with multiple binary logistic regression model, which entered sexual satisfaction as the outcome variable, smoking status as the predictor, and socio-demographic and clinical variables at once to adjust for the potential confounding effects of these socio-demographic and clinical factors. By using the sample of smokers and the same analytic procedures, two multiple binary logistic regression models (one included the level of smoking as the predictor, and the other included the level of nicotine dependence as the predictor) were established to further examine the relationships between the two variables and sexual satisfaction. We used odds ratios (ORs) and 95% confidence intervals (CIs) to quantify the associations between variables and sexual satisfaction. The statistical significance level was set at P < 0.05 (two-sided). SPSS software version 15.0 package (SPSS Inc, Chicago, IL) was used for all analyses.

## Results

The average age of the 480 MMT HDPs was 38.3 years (range: 21–59 years), and 31.0% were females. In total, 154 patients (32.1%) were dissatisfied with their sexual life and 459 patients (95.6%) were current smokers. Numbers of light, moderate, and heavy smokers were 117 (25.5%), 99 (21.6%), and 243 (52.9%), respectively. Numbers of smokers who were mildly, moderately, and severely dependent to nicotine were 102 (22.2%), 69 (15.0%), and 188 (62.7%), respectively. Detailed socio-demographic and clinical characteristics of the patient sample were shown in **Table 1**.

**Table 1:** Characteristics of methadone-maintained heroin-dependent patients, spitted by smoking status.

Variables		Total sample (n = 480)	Non-smokers (n = 21)	Smokers (n = 459)	χ2	P
Sex*	Male	331	7(33.3)	324(70.6)		
	Female	149	14(66.7)	135(29.4)	13.02	<0.001
Age (years)	≤39	260	14(66.7)	246(53.6)		
	>39	220	7(33.3)	213(46.4)	1.382	0.240
Education years	Primary school and below	53	2(9.52)	51(11.1)		
	Junior middle school	249	12(57.1)	237(51.6)		
	Senior high school and above	178	7(33.3)	171(37.3)	0.247	0.884
Marital status**	Married	267	18(85.7)	249(54.2)		
	Non-married	213	3(14.3)	210(47.8)	8.055	0.005
Employment	Yes	252	20(95.2)	232(50.5)		
	No	228	1(4.76)	227(49.5)	17.059	<0.001
Route of heroin administration	Smoking	75	6(28.6)	69(15.0)		
	Injection	405	15(71.4)	390(85.0)	2.792	0.095
Duration of heroin use (years)	≤10	280	7(33.3)	273(59.5)		
	>10	200	14(66.7)	186(40.5)	5.647	0.015
Methadone dosage (mg/d)	<70	259	4(19.0)	255(55.6)		
	≥70	221	17(81.0)	204(44.4)	10.773	0.001
MMT duration (months)	≤24	242	11(52.4)	231(50.3)		
	>24	238	10(47.6)	228(49.7)	0.034	0.854
Depressive symptoms	No	265	9(42.9)	256(55.8)		
	Yes	215	12(57.1)	203(44.2)	1.355	0.244
Sexual dissatisfaction	No	326	18(85.7)	308(67.1)		
	Yes	154	3(14.3)	151(32.9)	3.192	0.074

*Collected via self-report, confirmed by patients’ treating psychiatrists, who had performed the physical examination for patients.

**“Married” included married and remarried; “Non-married” included never-married, separated, cohabitating, divorced, and widowed.

The prevalence rates of sexual dissatisfaction were higher in current smokers than non-smokers (32.9% vs. 14.3%) with a borderline significant P value of 0.074. As shown in **Table 1**, compared to non-smokers, smokers were more likely to be males, be non-married, be unemployed, have a short duration of heroin use, and take a low dose of methadone. After adjusting for socio-demographic and clinical factors (**Table 2**), current smoking was significantly linked to sexual dissatisfaction (OR = 1.95, P = 0.026).

**Table 2:** Multiple binary logistic regression on the association between smoking and sexual dissatisfaction in methadone-maintained heroin-dependent patients, controlling for socio-demographic and clinical factors.

Variables		OR(95%CI)	P
Current smoking	No	1	
	Yes	1.95(1.06,3.60)	0.026
Sex*	Male	1	
	Female	0.39(0.23,0.65)	<0.001
Age (years)	≤39	1	
	>39	2.31(1.49,3.59)	<0.001
Education years	Primary school and below	1	
	Junior middle school	0.98(0.50,1.94)	0.963
	Senior high school and above	0.53(0.26,1.10)	0.087
Marital status**	Married	1	
	Non-married	1.18(0.91,1.52)	0.216
Employment	Yes	1	
	No	3.63(2.35,5.60)	<0.001
Route of heroin administration	Smoking	1	
	Injection	2.22(1.21,4.08)	0.011
Duration of heroin use (years)	≤10	1	
	>10	1.08(0.70,1.69)	0.72
Methadone dosage (mg/d)	<70	1	
	≥70	1.13(0.90,1.63)	0.087
MMT duration (months)	≤24	1	
	>24	1.35(0.87,2.10)	0.182
Depressive symptoms	No	1	
	Yes	2.58(1.67,4.00)	<0.001

*Collected via self-report, confirmed by patients’ treating psychiatrists, who had performed the physical examination for patients.

**“Married” included married and remarried; “Non-married” included never-married, separated, cohabitating, divorced, and widowed.

Socio-demographic and clinical characteristics of the 459 smokers were displayed in **Table 3**. Overall, rates of sexual dissatisfaction in smokers increased with levels of smoking (light: 23.9%, moderate: 28.3%, heavy: 39.1%; P = 0.008) and nicotine dependence (mild: 15.7%, moderate: 24.6%, severe: 41.6%; P < 0.001). After adjusting for socio-demographic and clinical factors (**Table 3**), both heavy smoking and severe nicotine dependence were significantly linked to sexual dissatisfaction (OR = 1.80, P = 0.025; OR = 3.27, P < 0.001).

**Table 3:** Characteristics of methadone-maintained heroin-dependent smokers, and multiple binary logistic regression on the associations between levels of smoking and nicotine dependence and sexual dissatisfaction.

Variables		Total sample (n = 459)	Sexual dissatisfaction (n = 151)	%	OR(95%CI)	P	OR(95%CI)	P
Level of smoking	Light	117	28	23.9	1		—	—
	Moderate	99	28	28.3	1.16(0.62,2.16)	0.641	—	—
	Heavy	243	95	39.1*	1.80(1.08,3.02)	0.025	—	—
Level of nicotine dependence	Mild	102	16	15.7	—	—	1	
	Moderate	69	17	24.6	—	—	1.39(0.60,3.25)	0.446
	Severe	288	118	41.0*	—	—	3.27(1.78,6.03)	<0.001
Sex**	Male	324	120	37.0	1		1	
	Female	135	31	23.0*	0.47(0.25,0.88)	0.019	0.41(0.23,0.70)	0.001
Age (years)	≤39	246	49	19.9	1		1	
	>39	213	102	47.9*	3.03(1.78,5.17)	<0.001	2.82(1.74,4.57)	<0.001
Education years	Primary school and below	51	19	37.3	1		1	
	Junior middle school	237	95	40.1	1.01(0.48,2.15)	0.978	1.27(0.64,2.54)	0.492
	Senior high school and above	171	37	21.6*	0.42(0.19,0.95)	0.037	0.63(0.30,1.33)	0.227
Marital status***	Married	249	74	29.7	1		1	
	Non-married	210	77	36.7	1.39(0.84,2.30)	0.203	1.62(1.02,2.57)	0.039
Employment	Yes	232	49	21.1	1		1	
	No	227	102	44.9*	2.96(1.82,4.81)	<0.001	3.40(2.15,5.36)	<0.001
Route of heroin administration	Smoking	69	19	27.5	1		1	
	Injection	390	132	33.8	2.38(1.17,4.83)	0.017	2.27(1.20,4.33)	0.012
Duration of heroin use (years)	≤10	273	84	30.8	1		1	
	>10	186	67	36.0	1.23(0.75,2.04)	0.394	1.24(0.78,1.98)	0.361
Methadone dosage (mg/d)	<70	255	71	27.8	1		1	
	≥70	204	80	39.2*	1.87(1.13,3.08)	0.015	1.13(0.72,1.76)	0.595
MMT duration (months)	≤24	231	70	30.3	1		1	
	>24	228	81	35.5	1.02(0.63,1.67)	0.923	1.49(0.95,2.35)	0.085
Depressive symptoms	No	256	54	21.1	1		1	
	Yes	203	97	47.8*	3.08(1.88,5.05)	<0.001	2.43(1.54,3.83)	<0.001

*Comparisons between subgroups, P < 0.05.

**Collected via self-report, confirmed by patients’ treating psychiatrists, who had performed the physical examination for patients.

***“Married” included married and remarried; “Non-married” included never-married, separated, cohabitating, divorced, and widowed.

## Discussion

To the best of our knowledge, this is the first large-scale study that is specifically designed to examine the association between smoking behavior and sexual dysfunction of Chinese HDPs receiving MMT. We found a 95.6% prevalence of current smoking in Chinese MMT patients, which is consistent with the high smoking rates among patients receiving ORT in other countries (87.2%–98.1%) (26, 28), suggesting that smoking is also a very common issue in HDPs of Chinese MMT clinics. Results of our multiple regression analysis revealed a 1.95 times risk of sexual dissatisfaction in heroin-dependent smokers than non-smokers of MMT clinics, replicating the higher risk of sexual dysfunction in smokers than non-smokers in the general population (5–9). This association was further strengthened by the significantly higher risk of sexual dissatisfaction in heavy than in light smokers (OR = 1.80) and in severely than in mildly nicotine-dependent smokers (OR = 3.27), after controlling for potential socio-demographic and clinical confounders.

Psychopharmacological studies have found that nicotine is a potent parasympathomimetic stimulant, which can stimulate nicotinic acetylcholine receptors in the central nervous system and promote the release of excitatory neurotransmitters (i.e., acetylcholine, serotonin, norepinephrine, and dopamine) and beta-endorphin, resulting in mood-altering and analgesic effects (38–40). There is convincing evidence that a large proportion of MMT patients, although they have stopped using heroin, are still suffering from prolonged withdrawal symptoms of heroin dependence such as depression and physical pain (27, 30). Accordingly, an empirical study has found that the main reason for smoking in heroin users was “to maintain pleasure” (41). Therefore, MMT patients may smoke to obtain nicotine to reduce or relieve withdrawal symptoms. This could explain the very high rate of smoking in MMT patients.

Although cigarette smoking has a potentially beneficial effect on the withdrawal symptoms of MMT HDPs, heavy metals in tobacco smoke can also lead to elevated levels of reproductive toxic chemicals in the blood, such as lead and cadmium (42). The accumulation of the two chemicals in the body would directly damage gonadal cells and tissues and cause sexual dysfunction (43, 44). The second possible mechanism underlying the smoking-sexual dissatisfaction is the sexual hormone imbalances resulting from the dysregulation of the hypothalamic–pituitary–gonadal (HPG) axis (45–47). For example, smoking may inhibit the ovarian function and lower the level of estrogen, which is related to the higher risk of female sexual dysfunction (5, 48). Third, smoking could impair the endothelial function of arterial vessels and promote the stiffness of genital vessels, resulting in alterations in the blood flow to genital organs (49, 50). This affects, for example, blood flow to the penis, which is critical for initiating an erection (8).

Findings from pharmacological studies have shown that methadone and other opioids could suppress the release of gonadal hormones *via* its inhibitory effect on HPG axis, including testosterone, which plays a critical role in maintaining sexual desire in both men and women (51–55). Therefore, in the clinical management of heroin addiction, sexual dysfunction is generally considered as one of the most common side effects associated with methadone treatment (25, 56), as confirmed by the significant association between a high dose of methadone and sexual dissatisfaction in **Table 3** of the present report. However, findings from recent studies and the current study indicate that sexual dysfunction is the result of multiple factors including methadone treatment, clinical variables and cigarette smoking (17, 22–25). In this study, we found not only the significant association between smoking and sexual dissatisfaction but also the greater risk of sexual dissatisfaction in heavy smokers (relative to light smokers) and severe nicotine-dependent smokers (relative to mild nicotine-dependent smokers); the latter finding is in line with the dose-response relationship between cigarette exposure and sexual dysfunction reported in the general population (5).

This study has some limitations. First, data of this study were collected cross-sectionally; therefore, the causality of the relationship between smoking and sexual dissatisfaction needs to be further examined in longitudinal studies. Second, we did not measure the levels of blood sex hormones and nicotine; so the biological mechanisms underlying the smoking-sexual function link could not be determined. Third, since this was a self-report questionnaire survey, the prevalence of sexual dissatisfaction might be underestimated due to social desirability bias, that is, patients are more likely to under-report “bad behaviors” such as sexual dissatisfaction. Fourth, some other potential factors associated with sexual dissatisfaction such as the relationship with sex partners, number of sex partners, organic diseases (i.e., vascular disease and Peyronie’s disease), and the use of antidepressants were not measured and controlled in our adjustment analyses. It is uncertain whether or not these factors would inﬂuence the association between smoking and sexual dissatisfaction. Fifth, our study only assessed the association of smoking with an overall indicator of sexual dysfunction, sexual dissatisfaction. No data are available on the associations of smoking with specific types of sexual dysfunction such as desire and arousal disorders. Further studies are warranted to examine these associations. Finally, given the significant sex difference in the prevalence of sexual dissatisfaction, it would be more informative to present results on the smoking-sexual dissatisfaction relationship by sex. However, multiple analyses according to sex are not feasible due to the very small sample size of female patients with sexual dissatisfaction (n = 31). In this case, we had to use a combined sample of male and female patients to examine the smoking-sexual dissatisfaction relationship. Such aggregate-level results might mask the sex difference in smoking-sexual dissatisfaction relationship. Large-scale studies are needed to test whether the smoking-sexual dissatisfaction association differs between male and female patients.

In China, smoking is often regarded as a normal and socially acceptable behavior (57); thus, Chinese psychiatric physicians and nurses of MMT clinics seldom advise their patients to quit smoking. This study demonstrated the significant relationship between smoking and sexual dissatisfaction in Chinese HDPs receiving MMT, as well as the higher likelihood of sexual dissatisfaction in heavy smokers and severely nicotine-dependent smokers. Our findings may suggest the importance of smoking cessation for the clinical management of sexual dysfunction in Chinese MMT clinics. It is necessary to conduct further interventional studies to examine whether quitting smoking can improve the sexual satisfaction of methadone-maintained patients.

## Ethics Statement

The study protocol was approved by the Ethical Review Board of Wuhan Mental Health Center. Declarations of anonymity and confidentiality had been made and all subjects provided written informed consent before the formal survey.

## Author Contributions

JL and B-LZ were responsible for the design of the study and interpretation of data, W-XX and B-LZ for the manuscript draft and statistical analysis, Y-MX for the data collection and critical revision of the manuscript, and W-XX for statistical consultation, data extraction, and processing. All authors reviewed the data and analysis, revised the manuscript, had full access to all of the data in the study and can take responsibility for the integrity of the data and the accuracy of the data analysis, and had authority over approval of final manuscript version and the decision to submit for publication.

## Funding

This study was funded by the Subject Leadership Training Programme for Medicine Discipline of Health and Family Planning Commission of Yunnan Province (D-2017048, JL, PI), Health and Family Planning Commission of Yunnan Province [2016NS027, JL, PI], and Wuhan Health and Family Planning Commission [WX17Q30, YM Xu, PI; WG16A02, BL Zhong, PI]. The funders had no role in study design, data collection and analysis, decision to publish, or preparation of the manuscript.

## Conflict of Interest Statement

The authors declare that the research was conducted in the absence of any commercial or financial relationships that could be construed as a potential conflict of interest.
